# A systematic review of frameworks for the interrelationships of mental health evidence and policy in low- and middle-income countries

**DOI:** 10.1186/s12961-018-0357-2

**Published:** 2018-08-22

**Authors:** Nicole Votruba, Alexandra Ziemann, Jonathan Grant, Graham Thornicroft

**Affiliations:** 1 Centre for Global Mental Health, Health Service and Population Research Department, Institute of Psychiatry, Psychology & Neuroscience (IoPPN), King’s College London, David Goldberg Centre Rm: M0.08 PO Box 28, De Crespigny Park - Denmark Hill, London, SE5 8AF United Kingdom; 2 Centre for Implementation Science, Health Service and Population Research Department, Institute of Psychiatry, Psychology & Neuroscience (IoPPN), King’s College London, David Goldberg Centre Rm: M0.08 PO Box 28, De Crespigny Park - Denmark Hill, London, SE5 8AF United Kingdom; 3 King’s Improvement Science at the Centre for Implementation Science, NIHR CLAHRC South London, Health Service and Population Research Department, Institute of Psychiatry, Psychology & Neuroscience (IoPPN), King’s College London, David Goldberg Centre Rm: M0.08 PO Box 28, De Crespigny Park - Denmark Hill, London, SE5 8AF United Kingdom; 4 Policy Institute at King’s, Virginia Woolf Building, The Strand, King’s College London, London, United Kingdom

**Keywords:** Evidence-informed policy-making, Knowledge translation, Research impact, Policy impact, Evidence-based policy, Research evidence, Mental health, Low- and middle-income countries, Theory review

## Abstract

**Background:**

The interrelationships between research evidence and policy-making are complex. Different theoretical frameworks exist to explain general evidence–policy interactions. One largely unexplored element of these interrelationships is how evidence interrelates with, and influences, policy/political agenda-setting. This review aims to identify the elements and processes of theories, frameworks and models on interrelationships of research evidence and health policy-making, with a focus on actionability and agenda-setting in the context of mental health in low- and middle-income countries (LMICs).

**Methods:**

A systematic review of theories was conducted based on the BeHeMOTh search method, using a tested and refined search strategy. Nine electronic databases and other relevant sources were searched for peer-reviewed and grey literature. Two reviewers screened the abstracts, reviewed full-text articles, extracted data and performed quality assessments. Analysis was based on a thematic analysis. The included papers had to present an actionable theoretical framework/model on evidence and policy interrelationships, such as knowledge translation or evidence-based policy, specifically target the agenda-setting process, focus on mental health, be from LMICs and published in English.

**Results:**

From 236 publications included in the full text analysis, no studies fully complied with our inclusion criteria. Widening the focus by leaving out ‘agenda-setting’, we included ten studies, four of which had unique conceptual frameworks focusing on mental health and LMICs but not agenda-setting. The four analysed frameworks confirmed research gaps from LMICs and mental health, and a lack of focus on agenda-setting. Frameworks and models from other health and policy areas provide interesting conceptual approaches and lessons with regards to agenda-setting.

**Conclusion:**

Our systematic review identified frameworks on evidence and policy interrelations that differ in their elements and processes. No framework fulfilled all inclusion criteria. Four actionable frameworks are applicable to mental health and LMICs, but none specifically target agenda-setting. We have identified agenda-setting as a research theory gap in the context of mental health knowledge translation in LMICs. Frameworks from other health/policy areas could offer lessons on agenda-setting and new approaches for creating policy impact for mental health and to tackle the translational gap in LMICs.

**Electronic supplementary material:**

The online version of this article (10.1186/s12961-018-0357-2) contains supplementary material, which is available to authorized users.

## Background

### The mental health evidence-to-policy gap

Mental disorders are among the most pressing health challenges of our time, both in terms of years of life lost and global burden of disability [1]. In low- and middle-income countries (LMICs), up to 85% of people with mental illness are untreated. One reason for this evidence-to-practice gap is to be found in the process of translation of evidence into policy [2]. Policies are essential for strengthening systems and services, and to overcome the mental health treatment gap [3]. However, translating evidence into policy in LMICs is difficult, particularly for mental health [4–6]. Theoretical and empirical research to guide action for knowledge translation and evidence-based policy-making in these contexts faces very specific challenges [6].

### Different understandings of research evidence and policy interrelations

Knowledge translation is a complex, lengthy and little understood process of push, pull, exchange and/or co-creation, aiming to make policy more evidence-informed [2, 7]. In health, evidence-based policy-making is generally seen as the gold standard, and both policy-makers and researchers widely claim to aim for evidence-based health policy-making [8]. Nevertheless, the realisation of this goal is limited by their political and academic realities [9]. This discrepancy has been coined as the ‘translational gap’ between evidence and policy-making [10, 11].

Many different concepts are in use to describe the process of interrelations and interactions between research evidence and policy [12–14]. Depending on the school of thought, academic field, theoretical concept, and which aims, determinants and outcomes of the process are in focus, these interrelations have been described as ‘knowledge translation’ [15], ‘knowledge transfer’ [13, 16], ‘knowledge transfer and exchange’ [17], ‘research uptake’ [18], ‘research utilisation’ [19], ‘evidence-based policy-making’ [20], ‘evidence-informed policy-making’ [21], or ‘translational research’ [22, 23], and more. Additionally, an overlooked area of the research–policy dynamic has been termed as ‘researcher utilisation’ [24]. A detailed summary on the different concepts of knowledge translation has been previously published [14, 25]. In this review, we aim to cover all of these concepts of evidence and policy interrelationships without giving a normative implication, and are therefore using the term ‘evidence and policy interrelationships’.

In the last decades, empirical and theoretical studies have increased in fields such as knowledge translation and exchange or evidence-based policy research to understand and reduce these translational gaps [9, 17, 26, 27]. A number of theoretical concepts and frameworks has emerged within health research [20], for different purposes, target groups and contexts, and for the translation of evidence into policy [28]. However, few theories are being tested against empirical works [29] and few studies link evidence with theories [30]. Recently, claims have been made that, overall, the translation of evidence to policy with existing theories has failed, that new inputs and innovative paradigms from other scientific areas are required [31], and that engagement with theories and approaches beyond the current remit of public health and knowledge utilisation is needed [29]. Increasing attention has been given to focusing on what type of evidence, how and under what circumstances policy-makers use research, but views from LMICs are needed [32].

### Context influences evidence and policy interrelationships

Context influences how evidence is perceived [33, 34]. Both empirical studies and conceptual frameworks should ideally consider that evidence into policy interrelations highly depend on contextual factors of country setting and the specific policy issue [35]. Herein, we focus on the context of LMICs and mental health.

#### LMIC context

Due to the high burden of disease and a lack of resources, the utilisation of research is particularly pertinent in LMICs [36]. Yet, evidence–policy interrelationships in LMIC settings differ from those in high-income countries [37]. Frequently, political contexts are troubled, policy-making processes are chaotic and obscure, research capacity is low, partnerships with policy are lacking, and donors and other policy elites, as well as an emerging civil society, are externally influencing the research-to-policy process [38, 39]. Therefore, the specific LMIC context requires appropriate and tailored strategies [40] and theoretical guidance for knowledge translation [41]. However, a vast research gap in these countries exists regarding what works for research evidence translation into mental health policy-making. Few theories have been empirically validated in the specific contexts of mental health and LMICs [9] or tested against case studies [42, 43]. More and better conceptual and practical research for understanding the complexities of LMICs, and how to bridge the evidence-to-policy gap are needed [38, 44].

#### The specific context of mental health

Despite the massive burden of disease, mental health is not a policy priority in LMICs [45]. Many countries in these settings do not have mental health policies, comprehensive plans or legislation in place [46, 47]. As a policy issue, mental health is highly complex, which is why the research–policy exchange is difficult and often fails for reasons such as stigma [48, 49], lack of political will [50], or insufficient knowledge capacity-building [51]. LMICs have higher risks of political instability, armed conflict, epidemics and disasters, all of which are critical determinants of mental disorders [47] and have a negative impact on the effectiveness of mental health evidence and policy exchange, thus reinforcing the low prioritisation of mental health in policy and research [52]. Additionally, human and financial resources are very limited, both for mental health services and research capacity [53], with little in-country research coming from LMICs on the interrelationships of research evidence and mental health policies [54]. Frequently, resources get distributed to more pressing (health) policy issues and are influenced by foreign aid policy prioritisation.

Mental health differs from other health and policy issues. Firstly, talking about mental health does not refer to one single policy issue, but rather comprises a heterogenous field of mental, behavioural or neurodevelopmental disorders with conditions ranging from depression and schizophrenia to autism and dementia [55]. This heterogeneity of globally differing conditions, causes and treatments, and the lack of a ‘one fits all solution’ has been identified as a leading factor for hindering mental health’s rise as a policy issue [56]. Secondly, there is a high prevalence of co-morbidity in mental health and physical illness, which needs to be appropriately addressed and requires a substantial change in health system, education and services [57]. Thirdly, beyond physical health, mental health is a cross-cutting issue with cross-sectoral impact. In order to put sustainable mental health care and treatment in place, there is rarely a single solution or treatment available. Instead, due to the existing disparities, frequently, a number of different sectors need to be addressed, ranging from social care, education, justice system, financing and even employment, gender equality or housing [58]. Finally, the overall field of mental health is fragmented, and does not unanimously agree on a clear approach to treatment and care. Despite the leading biomedical model represented in the diagnostic categories of the DSM-5 and ICD-10, globally conflicting views exist across psychology, psychiatry and neurology with regards to definitions, measurement, emphases and cross-cultural implementation [59, 60].

### A theory for evidence–policy interrelationships in mental health and LMICs

Considering context in evidence–policy interrelationships and designing a specific conceptual framework and systematic strategies can be helpful to understand and guide action [61]. Despite the distinct contextual challenges that mental health faces in LMICs as a policy issue, most knowledge translation studies rely on generic theories [62]. As Oliver et al. [9] demonstrated in continuation of earlier work [63], barriers to the translation of evidence into policy are enduring, and the application of models and theories from high-income settings has not sufficiently been tested on LMIC contexts. Additionally, recent research found that there is no unifying, predictive or actionable theoretical approach considering these complexities to increase the uptake of mental health evidence in policy, and that more systematic, rigorous strategies are required [62, 64].

Overall, policy-making is subject to numerous influences from interest groups, issue networks, social elites or ‘state level bureaucrats’ [65]. Intermediators for knowledge exchange, often referred to as ‘knowledge brokers’ or ‘policy entrepreneurs’ [66, 67], can play a role as enablers for the fragmented world of mental health policy-making; however, overall, their effectiveness remains unclear [68]. Research has stressed how actors within a policy triangle [69], cultural/value systems [70] and knowledge–power interactions [71], as well as context, time and specific policy issues [72] influence country-specific differences. Political and institutional mechanisms were found to be relevant influences on the use of evidence and decision-making [29]. Policy decision-making is complex and influenced by the (self-)interests of policy stakeholders and organisations [65]. Incremental policy-making models evolved but continue the linear view of decision-making, whereas ‘garbage can’ models focus on irrationality and unpredictability [73]. Calls for more research into deliberate and systematic strategies to enhance the research-to-policy gap have been made [61], and the different stages of the policy cycle and policy-making are similar in evidence-based policy-making and health research [74, 75]. Rather than fragmented approaches, or attempting to develop a one-size-fits-all model, more research is needed to develop and test frameworks and conceptual models with regionally tailored approaches [76, 77], and to understand why a given type of evidence is used by a specific audience [64], and in which way, at each specific stage of the policy cycle [20].

### How mental health research can gain policy-makers’ attention: agenda-setting

**Fig. 1:**
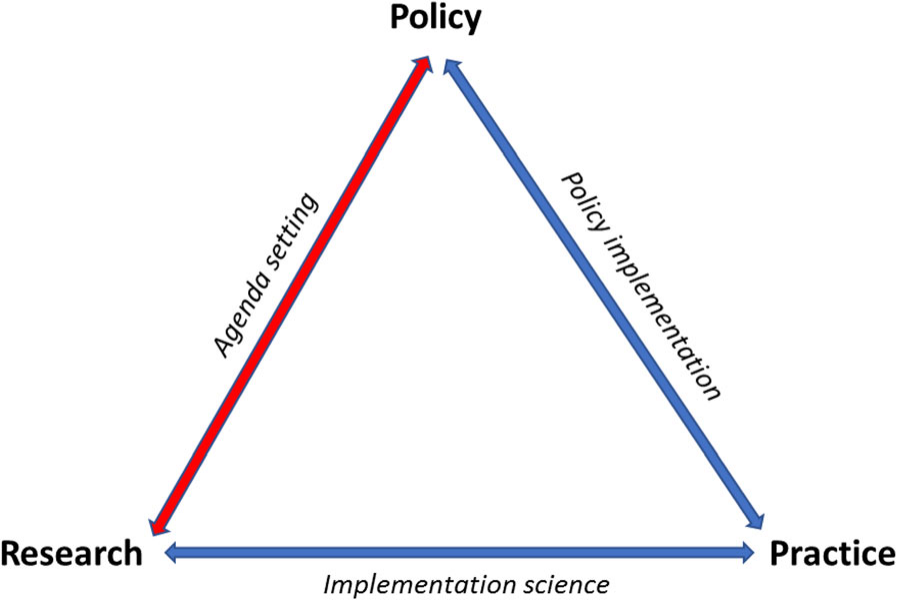
Simplified model of evidence into policy and practice processes (designed by authors)

Despite the striking evidence and huge treatment gap, mental health is not an issue on the national policy agenda in many LMICs. Equally, global development policies, such as the 2015 United Nations Sustainable Development Goals, do not stress mental health as a policy priority [78, 79]. Therefore, how can a research topic gain and maintain the attention of policy-makers? For research evidence to be effective in practice, it first needs to be taken up as a policy issue on the policy agenda and translated into policy (see model Fig. 1).

Agenda-setting, or policy priority-setting, occurs when a topic gains and maintains the attention of policy-makers [80]. In a very simplified model of a policy cycle, it is generally seen as the first stage followed by policy formulation [81]. More generally, agenda-setting involves identifying, defining and prioritising problems for policy attention [82], and provides a critical mechanism for the formulation, adoption and implementation of health policy [83]. Herein, we use the term ‘agenda-setting’ as a policy pre-decision-making process [84], partly overlapping with the process of policy formulation (and others), and use the expression synonymously to policy priority-setting. It can partly overlap with, but differs from, research process or outcome priority-setting [85].

The health policy field has been focusing on agenda-setting for considerable time, aiming to understand how and why issues gain policy priority [81, 86, 87]. However, much remains unclear, including why and when specific health issues arise as policy priorities on the policy agenda [88], how political will emerges and is sustained to take action, and how scientific research and policy decision-making interrelate [2]. The critical role of agenda-setting in getting mental health evidence on the policy agenda has been examined in an empirical policy analysis in Australia [89], and links have been investigated between agenda-setting/health policy research and knowledge translation in Canada and in LMICs [62, 90]. Overall, little research on this topic has focused on LMICs [70, 91], yet, a prevailing health policy agenda and the lack of political will have been identified as key barriers to adequate mental health service development in LMICs [50]. Others have stressed the relevance of agenda-setting for research on neglected health issues [36, 92], and it has even been claimed that research evidence can best influence policy-making at the agenda-setting and policy formation stages [93]. Despite this, in knowledge translation, agenda-setting is yet to appear as a focus point, and rather the two remain as two parallel streams. It has been recently claimed that using entry points according to the policy stages can be helpful to achieve greater policy traction for mental health [56].

### Hypothesis and aim

Following these claims for new paradigms [31, 56], we herein depart from the hypothesis that, for a critical policy issue like mental health in the context of LMICs, knowledge translation may be partly failing because agenda-setting is not specifically targeted and research evidence and policy interrelationships could potentially improve if theory-guided actions were focused on policy agenda-setting. Therefore, the research aim of this paper is to identify theories, frameworks and models to understand and guide action in research evidence and policy interrelationships in mental health and LMICs that target policy agenda-setting. Our main objective is to explore the elements and processes within these frameworks by using a qualitative systematic review of theories. To our knowledge, no such review has been previously performed or published.

## Methods

**Fig. 2:**
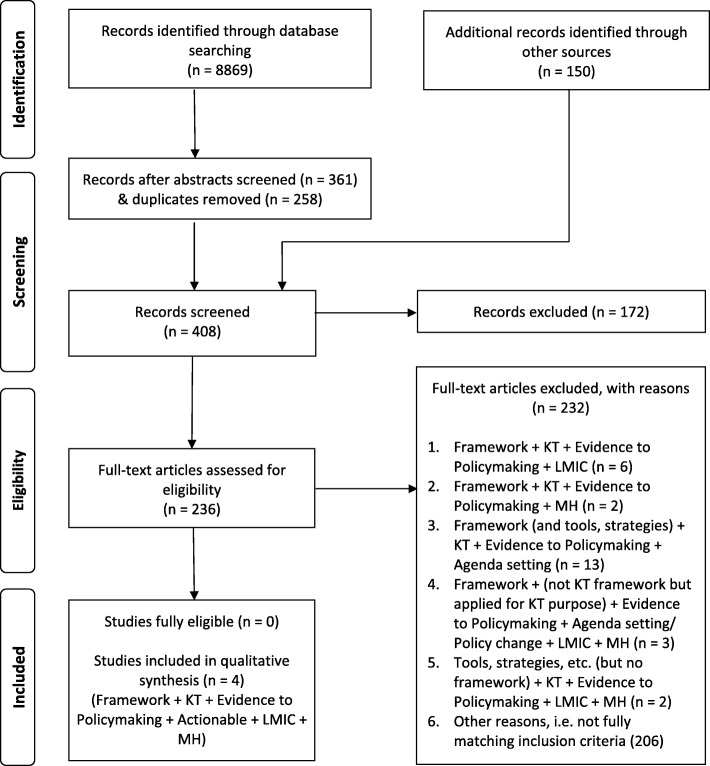
PRISMA Flow Diagram: Review of theories of research and policy interrelationships

We performed a qualitative, systematic review of theories. Systematic reviews of theories differ from systematic reviews of empirical data, and therefore methods need adaption; however, few guidelines exist [94, 95]. We applied a novel, structured method to identify theoretical frameworks, named ‘theory review’ [96]. Throughout the search, appraisal and analysis, we followed the BeHEMoTh procedure. Additionally, we adapted and applied the PRISMA flow chart for the systematic review process [97] (Fig. 2).

### Search strategy

The search strategy was refined and adopted in several iterative steps. The suitability of the search strategy, process, criteria and quality appraisal was discussed in the group [94].

A first narrow search strategy that included qualifying search terms such as ‘mental health’ and low- and middle-income countr*’ returned very few results and was thus expanded to a more exploratory search with simpler, more flexible keywords [42, 98] such as (*health policy* AND (policy mak* OR decision mak*) AND (theor* OR *model* OR *framework* OR *concept*) AND (*evidence* OR *research* OR *knowledge*) AND (*translation* OR *transfer* OR *uptake* OR broker*). Searches were carried out in the following nine databases: Embase, Global Health, JSTOR, Medline, PsychINFO, PubMed, ScienceDirect, Scopus and Web of Science. In addition, grey literature was searched on websites of organisations working in the field (such as World Health Organisation (WHO), Overseas Development Institute (ODI), RAND, United Kingdom government), reference lists of identified studies were scanned and a number of relevant articles was identified through hand search and forwards and backwards citation tracking [94]. We followed an iterative, pragmatic approach that has been recommended as an effective method for theory reviews to identify additional studies providing relevant information rather than keyword search alone, which has been found inadequate for theory identification [99, 100].

To define and guide our final search strategy, we applied, adapted and followed the BeHEMoTh template for systematic identification of theory, defining behaviour of interest (Be), health context (H), exclusions (E), and models/theories (MoTh) (Table 1) [96], which consists of four steps, as follows:

Step 1: According to the BeHEMoTh strategy, we identified incidental occurrences of theory in our internal databases to inform our searches (step 1a), searched databases combining ‘behaviour of interest and health context’ with generic theory-related terms (step 1b), and we searched by dropping a concept from the BeHEMoTh search (mental health and LMICs) (step 1c).
Step 2: We compiled a list of named theories and merged it with our findings.
Step 3: For relevant articles we performed a phrase search for identified theory names combined with either behaviour of interest or health context.
Step 4: We identified key citations for frameworks that were earlier identified in Step 2, combining key source citations with behaviour of interest or health context. This step helped to retrieve theories that are not named in the abstract but occur in the reference lists and would otherwise be hidden [96].

**Table 1:** BeHEMoTh framework adapted to this systematic literature search

Be:	Behaviour of interest	Evidence and policy interrelations/interactions, i.e. knowledge translation, evidence-based policy-making, knowledge brokering, linkage and exchange, evidence-informed decision-making
He:	Health context	Health research, policy-making, mental health, LMICs
E:	Exclusions	Exclude non-theoretical models
MoTh:	Models or Theories	model* or theor* or concept* or framework*

### Inclusion criteria

Abstracts were screened and included according to the inclusion criteria outlined in Table 2.

**Table 2:** Literature inclusion and exclusion criteria

Criteria	Inclusion criteria	Exclusion criteria
Framework	Clearly describe the framework, i.e. describe process, determinants, strategies	No clear description of framework/theory/model, only mentions; only describes elements/parts/tools
Knowledge translation	Framework is used for knowledge translation (also described as the process of evidence-based policy-making/decision-making, etc.)	No evidence and policy interrelationship, knowledge translation, evidence-based policy-making/decision-making, policy change
Scientific research evidence	Relates to scientific/research knowledge	Does not explicitly relate to scientific/research knowledge, e.g. not tacit knowledge, user group or patient knowledge
Evidence to policy-making process	Must focus on the evidence into policy-making process (i.e. interactions between researchers and policy-makers)	Does not or only very vaguely describes the evidence to policy process; focuses on policy implementation into practice process
Agenda-setting and policy formulation	Aims at/includes process to agenda-setting (and/or policy formulation)	Only describes the process of informing policy
Action framework	Capable of guiding researchers in developing, applying and testing of knowledge translation interventions. We understand actionable as providing conceptual clarity, having a clear purpose, being able to explain how individuals move from intention to actual behaviour change, and useful to develop and test interventions [55, 56]	Not actionable, descriptive model, only part/components of the process
LMICs	Developed for/applied to LMICs, as defined by World Bank classification [148]	Only developed for/applied to high-income countries
Mental health	Developed for/applied to mental health	Only applied to physical health
Health	On human health	Not on human health
Language	Publication language: English, and accessible online as full article or retrievable as hard copy	Not in English, not retrievable
Publication date	No date limitation	n.a.
Study type	No study type restriction (also incl. reviews and case studies included)	n.a.

*n.a.* not applicable, *LMICs* low- and middle-income countries

We moved from other theoretical differentiations [101, 102] to the concept of ‘actionable framework’, which we found most suitable for our search aim for a framework to guide actions. We understand ‘actionable’ as providing conceptual clarity, having a clear purpose, being able to explain how individuals move from intention to actual behaviour change, and useful to develop and test interventions [103, 104].

We excluded articles without a clear description, or only parts, of a framework/theory/model, without relation to knowledge translation or research evidence, from outside the health field, if they were developed for/applied to high-income countries, and if they only vaguely described the process of informing policy. We also excluded frameworks that were not clearly actionable.

### Study selection

A first reviewer (NV) assessed abstracts, and obtained and assessed full texts where they seemed to meet the inclusion criteria. A second reviewer (AZ) screened abstracts in a random sample of 20%. Unclear cases were discussed and agreed upon with the second reviewer (AZ) for 100% inter-rater agreement. We only included key publications with full descriptions of the framework, not evaluations/applications. Duplicates were removed and studies describing the same framework were combined into unique studies.

### Quality appraisal

There is no comparable equivalent for quality appraisal of theoretical studies as there is for other study types such as the PRISMA checklist [105, 106], PICO for clinical evidence [107], or CASP [108] or SPIDER for qualitative reviews [94, 109]. Therefore, we followed a more inductive, subjective approach that has been recommended for use instead of checklists [94]. Practical ‘quality assessment prompts’ were found most suitable and were applied to ensure clear aims and objectives, a clearly specified and appropriate research design, clear account of reproduction of their findings, sufficient data to support their interpretations, and appropriate/adequately explicated analysis [110]. Methodological quality appraisal was performed alongside data extraction [111].

A second reviewer (AZ) checked that relevance appraisal criteria were consistently met in random samples of 20% of included papers, and disagreements were resolved through discussion for 100% agreement [94]. Studies varied greatly in quality and details of the described theory/framework/model, which caused difficulties in assessing their relevance [111]. Appraisal for conceptual distinction and applicability of theories, models and frameworks was challenging.

### Data extraction

Data extraction was guided by our research aims. The first reviewer critically assessed the literature and extracted details of theories, frameworks and models of evidence and policy interrelationships into a coding framework (excel spreadsheet) according to the following scheme: inclusion criteria, framework name and short description (the full data extraction sheet can be obtained upon request from the authors).

### Data analysis and synthesis

We analysed the data in an iterative multiple-stage process (iterative text analysis), reading the description of the framework, assessing it for the general inclusion criteria (framework/theory/model, knowledge translation, health, research evidence), going back and revisiting the frameworks for more specific evaluation (research into policy, agenda-setting, actionable), and eventually comparing and assessing the frameworks for the more specific inclusion criteria (mental health, LMICs) [94]. Through this inductive process, using thematic analysis, we were able to arrange key concepts and themes emerging from the frameworks (subjective induction), without excluding relevant frameworks too early. In the narrative theory synthesis, we identified and grouped similar theoretical concepts across the literature [95].

## Results

Through the database search we identified 8869 articles whose titles and abstracts were screened. We included 258 articles for full-text screening and another 150 articles were found through reference lists of relevant articles, grey literature and the BeHEMoTh procedure. After the second screening, another 172 records were excluded and 236 full-texts were assessed for eligibility. We found no (*n* = 0) frameworks that fully met all our inclusion criteria, including a clear focus on agenda-setting. We therefore amended our inclusion criteria to drop ‘agenda-setting’. Through this, we identified 10 papers presenting altogether four unique frameworks [56, 64, 112–119] that met our inclusion criteria, except that they were not specifically targeting agenda-setting. We included these four frameworks in the qualitative analysis and synthesis, and to identify potential agenda-setting elements. See Fig. 2 (PRISMA Flow chart) for the process of searching and screening for inclusion.

We developed a table for qualitative analysis and synthesis of the frameworks (Table 3). We also identified 20 other papers that matched some of the inclusion criteria but not all, so they were not included in the thematic analysis. An overview of the frameworks and references can be found in Additional file 1: Overview of categories with publication and framework details.

**Table 3:** Included frameworks and common themes (showing the elements identified in the frameworks and the themes derived from them by the authors)

Name	Theme identified	1. RAPID	2. KPP	3. SPIRIT	4. Country-level assessment
Key publication		Overseas Development Institute, 2004 [116]	Jones et al., 2009 [114]	Redman et al., 2015 [117]	Lavis et al., 2006 [115]
Elements	Political context	Political context: politics and institutions	Political context	Policy influences	General climate
	External influences	External influences: socio-economic and cultural influences, donor policies	Sectoral dynamics		
	Actors		Actors’ interests, values and beliefs		
	Evidence	Evidence: credibility and communication	Types of knowledge	Reservoir of relevant and reliable research	Production of research
				Research use: conceptual, instrumental, tactical and imposed fashions and to support policy agenda-setting, policy development, implementation or evaluation	Evaluation
					Efforts to facilitate user pull (Analysis: also partly in theme Catalysts)
	Intermediaries and links	Links: influence and legitimacy Media, advocacy, networking	Knowledge intermediaries		Exchange efforts
	Capacity		Capacity-building	Capacity	Push efforts
				Research engagement action: Agency to access and appraise research findings, commission or undertake research to generate new findings, or interact with researchers	User-pull efforts
	Catalysts			Catalysts: occurs to initiate the process of engaging with or using research	Efforts to facilitate user pull (Analysis: also partly in themes Evidence and Intermediaries and links)
	Other frameworks		Innovative frameworks (embed within an understanding of the broader system in which they work, and the relationship between the supply of and demand for knowledge on development policy issues)		

*KPP* Knowledge, policy and power framework, *RAPID* Context, evidence, links framework, *SPIRIT* SPIRIT Action Framework

### Characteristics of the included frameworks

We included four frameworks in the thematic analysis, namely the Context, Evidence, Links framework (RAPID) [112, 116], the Knowledge, Policy and Power framework (KPP) [114, 119], the SPIRIT Action framework (SPIRIT) [117], and the Framework for assessing country-level efforts to link research to action (Country-level framework) [115] (Table 3).

We assessed the quality of the studies and found differences in the four frameworks. The SPIRIT framework development gave a very explicit methodology [117], while the RAPID and KPP framework were developed based on “*theoretical, case study and practical work*” of the collaborating researchers/organisation (ODI) [119]. Similiarly, the Country-level framework is grounded in the authors’ earlier work [120], and acknowledges that much of it is only indirectly based on research, stating the research gap as a reason [115]. Overall, we found the methodological quality of theory development to be largely insufficiently clarified, which is a limitation to the interpretation of these results.

### Origins of the frameworks

The four frameworks included have all been developed (first published) between 2004 and 2015. All frameworks were developed (led) by researchers from high-income countries, and it was only clear for one framework that researchers from LMICs were involved in the development (Country-level framework [115]). Two frameworks (RAPID [116], KPP [114]) have been developed in the United Kingdom, by an international development organisation (ODI) and have been described as developed based on their longstanding empirical experiences in LMICs. Further, while KPP is being described as based on the lessons working with the RAPID framework, the authors do not see it as a next generation or substitute for the earlier RAPID framework (this was confirmed in discussion with one of the authors). One framework (SPIRIT [117]) has been developed by a study team from Australia and the United Kingdom, led by a not-for-profit organisation specialised in promoting the use of research evidence in health policy (Sax Institute), and involving policy-makers, researchers and knowledge exchange specialists. One framework (Country-level framework [115]) has been developed by a research collaboration from Canada, Malaysia and Uganda.

### Common themes identified in the frameworks

In our analysis, we synthesised the frameworks and their components for better comparison and identified seven relevant themes, namely political context, external influences, actors, evidence, intermediaries and links, capacity, and catalysts. While the elements and processes differ between the frameworks, these themes were common throughout.

1. *Political context*: All frameworks describe political context, politics and institutions, and policy influences as a unique, relevant component; only the country-level framework includes this more broadly in its element ‘general climate’ [115].
2. *External influences*: Two frameworks explicitly describe external influences such as socioeconomic and cultural influences and donor policies [112], or sectoral dynamics [114].
3. *Actors*: Only one framework has a single analytical component on actors’ beliefs, values and interests [119], an element which is only implicitly included in the other three frameworks. Other frameworks include actors in other elements such as ‘political context’, ‘external influences’ or ‘intermediaries and links’ [112], or only very implicitly [115, 117]. Actors can take the role of an agent of change, but are not necessarily considered as such.
4. *Evidence*: All four frameworks consider research evidence as in types of knowledge [119] or its credibility and communication [116]. One framework splits evidence into two parts, the origin (reservoir of relevant and reliable research) or the way (conceptual, instrumental, imposed) and purpose it is being used in or for [117]. The Country-level framework separates production of research and evaluation, and to some extent also the accessibility of research in efforts to facilitate user pull [115].
5. *Intermediaries and links*: Three of the frameworks stress elements of linkage, such as knowledge intermediaries [119] or communities, networks and intermediaries that hold legitimacy with the ability to influence [116], or as exchange efforts. Elements can also be found in efforts to facilitate user pull [115]. Intermediaries and links are agents and drivers of change.
6. *Capacity*: Two frameworks clearly consider an element of capacity. One framework splits this into capacity within the organisations (both researchers and policy-makers) and research engagement actions that enable the agencies to access and appraise research findings, commission or undertake research to generate new findings, or interact with researchers [117]. Capacity can also be identified in user-pull efforts and push efforts in the country-level framework [115]. Capacity is not included in the RAPID framework [112], while the KPP framework clearly stressed capacity-building in the first publication [114], but did not keep this framework element in later publications [119].
7. *Catalysts*: One framework clearly states the element of catalysts as incidents, actions or events occurring to initiate the process of engaging with or using research [117]. Other frameworks are less explicit about this element; however, it does occur elsewhere in efforts to facilitate user pull [115].
8. *Other frameworks:* One framework mentions, in the earlier publication [114], the application of innovative frameworks within the knowledge translation framework. However, this element has not been picked up since in later publications of the framework [119], or in any of the other frameworks.

### Application of the frameworks to the research aim

The research aim was to identify frameworks that can understand and guide actions (actionable) for mental health evidence into policy translation in LMICs and that target the specific challenge of policy agenda-setting. In the following sections we will discuss communalities and differences identified when we analysed the frameworks according to our research aim.

#### Different understandings of research evidence to policy-making

We found different understandings of research evidence to policy-making, knowledge translation and evidence to policy-making. According to the inclusion criteria, all frameworks focused on research evidence. Two frameworks (RAPID and KPP) describe knowledge translation as a ‘research push’, implying as coming from the perspective of, or specifically targeting, researchers, non-governmental organisations (NGOs) or other individuals or groups interested in engaging in the evidence push to policy-making [112, 119]. One framework (SPIRIT) focuses on the perspective of research uptake and describes the target group for intervention as the policy organisation [117]. One framework (Country-level framework) takes the perspective of knowledge bridging and targets governments on the organisational/systems level to enable research linkage and exchange [115].

#### Actionable frameworks

All four frameworks were described by the authors, and evaluated herein, as actionable. We understand a framework as ‘actionable’ if it can guide action (1) for researchers to translate evidence into policy, (2) for policy-makers to pick up research, and (3) for country-level planning to initiate linkage and exchange between research and policy-making. One framework (SPIRIT) clearly states the purpose of being developed to guide action for agencies to improve the use of research in their work [117]. The RAPID framework encompasses a simple analytical framework and practical tools for researchers to take action [116]. The KPP framework is described as a practical framework to analyse the knowledge–policy interface, rather than guiding action, but includes practical suggestions for promoting change and help to identify concrete, practical actions [119]. The main purpose of the Country-level framework is described as to inform dialogues to link research to action [115].

#### Agenda-setting

None of the four frameworks specifically targets the agenda-setting stage. However, the SPIRIT framework states a clear agenda-setting element as part of their outcome element, and acknowledges that research will be used to support policy agenda-setting (as well as policy development, implementation or evaluation) [117].

#### Application of the frameworks on mental health and LMICs

None of the four frameworks has been developed specifically for the purpose of mental health knowledge translation in LMICs, yet all frameworks were later applied by other researchers/authors to that specific context of mental health and LMICs. The RAPID framework was first applied to the context of mental health in 2006 in Vietnam [113]. The KPP framework was first used in 2014 in the United Kingdom to analyse global mental health policy-making context and networks (but not applied to a country setting yet) [56]. The SPIRIT framework was used shortly after its publication in 2015 as a structural framework in a systematic analysis in mental health and LMICs, however it has not yet been used in an empirical study [64]. The Country-level efforts framework was applied in 2015 by a research group in Lebanon [118].

## Discussion

In this study, we performed a systematic literature review to identify actionable frameworks on knowledge translation of research evidence into policy that specifically targeted agenda-setting, focusing on mental health in LMICs. No framework was found to fully comply with all inclusion criteria, but we identified and included four frameworks that complied with all inclusion criteria except for targeting agenda-setting. We identified different elements that were consistent within the compared frameworks, namely political context, external influences, actors, evidence, intermediaries and links, capacity, and catalysts.

### Relevance of the findings

Our findings are surprising and interesting on several levels. The frameworks confirmed different conceptual understandings of evidence into policy-making, as well as stereotypes on mental health research in LMICs, such as underrepresentation and research gap. However, we found that there could be an indication for increasing research in mental health knowledge translation in LMICs. Agenda-setting was not found to be a focus in frameworks for evidence and policy interrelationships.

#### Different conceptual understandings and approaches of evidence into policy-making

The frameworks had a different focus on knowledge translation and aims and targeted different groups. Two targeted researchers, NGOs or other individuals or groups for research push, one focused on the research uptake at the policy organisation, and one targeted government research linkage and exchange activities at the systems level. This confirms the variety of different approaches and concepts found in the literature with regards to the purpose and timing, how, what evidence is interacting and how, and how it is transferred, utilised, translated, picked up, exchanged, linked or facilitated to policy-making [2, 22, 121].

#### Non-academic research focus and research gap from LMICs

Interestingly, the majority of included frameworks were developed by NGOs or research collaborations led by NGOs such as development organisations (ODI) or think tanks (Sax Institute). This indicates that non-academic research provides strong contributions to the evidence base of knowledge translation and exchange, which might, however, not always be visible and accessible to researchers and implementers from academia.

Our findings also confirmed a research gap from LMICs and the bias that arises with it. Even though all included frameworks were developed by experienced global health collaborations, they were all led by researchers from high-income countries (United Kingdom, Canada, Australia), and only one framework was co-authored by researchers from LMICs [115]. Knowledge translation in mental health in LMICs remains biased as a research topic [122], and our findings confirm a need for addressing this gap in research capacity in LMICs. However, overall, the situation seems to be changing, as we found indication in our study for an increase in research on knowledge translation and evidence-to-policy frameworks in LMICs (six studies, published between 2006 and 2016).

#### Is mental health evidence into policy translation growing?

Interestingly, all included frameworks have been developed in the past 15 years, with three of them having been first applied in the past 4 years. This could indicate a growing attention for knowledge translation in mental health in LMICs and an increased theoretical interest and engagement in the topic of mental health policy-making. In many LMICs, mental health is barely, or not at all, a policy issue, and our findings appear to have identified a research gap and potential impact gap for knowledge translation efforts. A definition for ‘research impact’ has been proposed recently for (mental) health policy [12].

#### Agenda-setting is not a focus in knowledge translation

We were surprised to find that only one of the frameworks (SPIRIT) has a defined agenda-setting purpose within their element ‘outcome’, and see an aim of research to be used to support policy agenda-setting (and other policy-making stages) [117]. This is more understandable, considering that the interrelationships of evidence and health policy-making are complex and little understood, as well as the specific influence of research on the agenda-setting stage in the policy decision-making process [2]. Entry at the agenda-setting stage is most often a prerequisite for any topic for further policy discussions, decisions and implementation (Fig. 1). Even though agenda-setting (together with the stage of policy formation) has been found to be the best way to influence policy-making [93], it is surprising that few frameworks seem to consider this specific stage and rather describe wider concepts and actions for knowledge translation.

Interestingly, outside of the evidence-based policy-making and knowledge translation fields, agenda-setting has been a focus of research in other disciplines, particularly in health policy research. It seems that developments of frameworks for agenda-setting and policy change have been developed largely in separate streams, but some of them have also been repeatedly applied to evidence and policy interrelationships such as the Advocacy Coalition Framework [123] or Kingdon’s policy agenda-setting framework [93].

### Research in context

While our framework review was very narrow and specific, a number of other conceptual models and frameworks to describe, understand or guide the process of (or parts of) knowledge translation provide interesting contextual considerations, innovative approaches and potential guidance for mental health evidence and policy interrelationships in LMICs and agenda-setting in health policy-making.

#### Frameworks on evidence to policy-making, agenda-setting and LMICs

A number of frameworks outside the mental health field provide interesting approaches for evidence–policy relations and agenda-setting in LMICs. An interesting action model to create windows of opportunity for policy change from Kenya translates action through agenda-setting, coalition building and policy learning [90]. A study from South Africa on maternal health care combined a knowledge translation framework [80] and a policy agenda-setting framework [93] for windows of change [36]. Researchers from Lebanon developed a conceptual framework for a backward design in knowledge translation, that considers both priority-setting and capacity-building as key elements for evidence-informed policies [21]. A proposed conceptual framework highlights the relevance of entry points for policy issues in LMICs [124].

Others have more widely worked on structural and process changes to improve evidence and policy interrelations in LMICs. Embeddedness was expanded as a key structure for translating health policy and systems research into policy in LMICs [26]. In Nigeria, a research policy group based their evidence-informed policy-making strategies model on directly engaging policy-makers to increase their use of and capacity to use research [125].

Learnings can also be drawn from other public health issues with which mental health shares a number of commonalities, and which have recently gained traction on the policy agenda in LMICs, such as HIV/AIDS [126], malaria [127], tuberculosis [128] or maternal deaths [129].

#### Frameworks on evidence to policy-making relating to mental health

Several interesting approaches to improve evidence–policy relationships for mental health have been developed in or for high-income countries. A mental health policy group in Canada adopted an existing model on deliberative dialogues [130] to make the consensus-building process meaningful, and found this to be particularly effective in consensus conferences [131]. Another model from Canada builds on linkage and exchange at the organisational level aiming at policy formation [132]. Although these approaches are not for LMICs and do not include agenda-setting, a strong emphasis on interaction and exchange can be taken as a lesson for mental health policy-making in other contexts.

#### Frameworks, tools and strategies for evidence–policy interrelationships with a focus on agenda-setting

A number of conceptual models for evidence–policy interrelationships with a focus on agenda-setting evolved outside the context of mental health and LMICs. The elaborate stages of assessment of research utilisation in the interfaces and receptors model by Hanney et al. [73] was developed further into pathways to the use of health services research in policy by Gold [133]. A number of tools and strategies were developed to improve research uptake [24, 134, 135], but increasingly the focus is on co-production [136], and push, pull and exchange [137]. A planned project looks at information exchange networks in Canada [138], while others focus on specific strategies such as deliberative models [139], and others build models around context in evidence utilisation [33, 40]. Several strategies focused on specific research areas, such as enhancing the use of health systems research for health sector reform [140], an issue-based framework for health services research [80], or economic evaluations [141]. These frameworks provide interesting general models for linking for evidence–policy interrelationships and agenda-setting.

#### Frameworks from other fields, applied for evidence and policy interrelationships, focusing on agenda-setting and policy change for mental health in LMIC

A number of frameworks emerged in other fields, such as health policy research, focusing on agenda-setting and/or policy change for mental health in LMICs and were (indirectly) applied for evidence and policy interrelationships. In particular, the Advocacy Coalition Framework has been frequently applied to the health policy context in LMICs [123]. Another relevant conceptual approach was developed based on a combination of the widely used Kingdon’s Policy Agenda-setting Framework [93] and factors affecting mental health policy-making [142]. The Interdisciplinary Research Framework for Multisectoral Mental Health Policy Development focuses on the researchers’ agenda and links policy problems to goals and specific sectors [143]. Although this was found to be a very interesting and relevant study, it does not specifically guide the creation, translation, linkage and exchange process of research, and has not yet been applied to the LMIC context. A number of studies have analysed the mental health policy process in LMICs, such as a study in Cambodia that developed a mental health policy analysis concept map [144] based on models by Walt and Gilson [69] and Reich [86].

#### Tools and strategies for evidence and policy interrelationships for mental health in LMICs

In addition, a number of tools and strategies for evidence and policy-making for mental health in LMICs have been developed. Although they are not conceptual frameworks or models, they provide very actionable approaches for knowledge translation efforts. The Global Mental Health Policy Toolkit provides very practical, actionable tasks for teams of any background to map, influence, link and access the policy interface [145]. Additionally, the SPIRIT project gives detailed strategies for implementation with tools (SEER, ORACLE, SAGE) [117]. Other authors focus on setting priorities for research, linking them with principles for context-driven, intersectoral and integrative approaches to change policy and systems in LMICs [146].

These models can provide additional lessons and guide actionable processes of knowledge translation in mental health policy-making in LMICs. Some of them offer conceptual approaches for targeting agenda-setting in order to change policy, although agenda-setting has not been a focus of traditional knowledge translation frameworks in mental health. Applying a focus on agenda-setting to mental health could be helpful in improving evidence and policy interrelationships in LMICs. Studies from other health fields that have done so could provide insights on how agenda-setting could be targeted and might be adapted to mental health.

### Limitations

There are limitations and strengths to this study. Firstly, we were looking for an actionable framework and our definition and interpretation of ‘actionable’ is an attempt for more impact, but it may not be the only possible approach to an applicable, impact-aiming framework for evidence into policy translation and exchange efforts. Indeed, many discussions were held within the research team to clarify our understandings of the concepts used, and on inclusion or exclusion criteria. Therefore, it is likely that we may have excluded frameworks that might be applicable after all.

Secondly, we were looking for frameworks focusing on evidence and policy interrelationships from a knowledge translation/evidence-based policy-making perspective to expand the field. However, there are potentially relevant studies from other fields on agenda-/priority-setting and policy change that also cover research evidence-to-policy processes in mental health and LMICs that our search may have missed due to the inclusion criteria.

Thirdly, the methodological quality of the frameworks was largely clarified. One framework had a very clear methodological base, but three of four frameworks were developed based on theoretical, case study and practical work of the collaborating researchers/organisations. While this may be related to the overall research gap, it represents a limitation to the interpretation of these results and shows that more research is needed to link and test strong methodological concepts with empirical research.

Fourthly, although we ran a very comprehensive search strategy in nine of the most relevant health databases without any publication time limit for the search, we may have missed some relevant studies. Only studies in English language were included, and therefore we missed at least one relevant-appearing publication in Portuguese [147], and others are likely.

One of the study’s strengths is the very strong methodological approach by applying the systematic BeHEMoTh strategy, which was found to be time-consuming, but to provide useful and rare guidance. We had a number of trial runs and discussions to refine the search strategy, and decided to keep the search terms broad, and scanned reference lists of relevant articles. The results of our grey literature search were quite substantial (*n* = 150), suggesting that this search was quite successful in identifying potentially relevant studies. However, it is still likely that we may have missed relevant work on conceptual frameworks, especially work published in the grey literature.

## Conclusion

While there is a great variety of conceptual models on knowledge translation and evidence-based policy-making, very few frameworks are actionable and have been applied to the context of mental health and LMICs. The interrelationships of research evidence and policy-making in mental health and LMICs are extremely complex, and empirical studies show that, in LMICs, these interrelationships often barely exist in the first place. It is likely that knowledge exchange efforts in the specific context of mental health and LMICs could be more effective if they considered and targeted the agenda-setting stage for getting research into policy. We performed a systematic literature review to identify actionable frameworks on knowledge translation of research evidence into policy in mental health in LMICs that specifically targeted agenda-setting. Four actionable frameworks were applicable on knowledge translation of research evidence into policy in mental health in LMICs but none of them specifically targeted agenda-setting. Although agenda-setting in itself is not a new area, our review has identified it as a theory gap in the specific context of mental health knowledge translation in LMICs, and it might provide a new focus point for theories of evidence and policy interrelationships that aim for impact. Exploring frameworks and models from other health areas and the policy field could provide interesting lessons for evidence and policy interrelationships on agenda-setting and creating policy impact for mental health in LMICs.

## Additional file

Additional file 1: Overview of categories with publication and framework details. (DOCX 38 kb)

## Abbreviations

BeHEMoTH: BeHEMoTh framework for systematic identification of theory, defining behaviour of interest, health context, exclusions and models/theories
LMICs: Low- and middle-income countries
NGO: Non-governmental organisation
ODI: Overseas Development Institute
WHO: World Health Organisation
