# Supplementary material for: A systematic review of frameworks for the interrelationships of mental health evidence and policy in low- and middle-income countries
Source: Health Res Policy Syst. 2018 Aug 22;16:85. doi: 10.1186/s12961-018-0357-2 (PMC6106735; doi:10.1186/s12961-018-0357-2)
Supplement: Supplementary file 1 — Overview of categories with publication and framework details. (DOCX 38 kb) [file 12961_2018_357_MOESM1_ESM.docx]

**Additional file 1: Overview of categories with publication and framework details**

| **Group 1** | **Group 2** | | | **Group 3** | | | **Group 4** | | | **Group 5** | | | **Group 6** | | | **Group 7** | | |
| --- | --- | --- | --- | --- | --- | --- | --- | --- | --- | --- | --- | --- | --- | --- | --- | --- | --- | --- |
| **Framework + KT + Evidence to Policymaking + Agenda setting + Actionable + LMIC + MH** | **Framework + KT + Evidence to Policymaking + Actionable + LMIC + MH** | | | **Framework + KT + Evidence to Policymaking + LMIC** | | | **Framework + KT + Evidence to Policymaking + MH** | | | **Framework + KT + Evidence to Policymaking + Agenda setting** | | | **Framework + (KT purpose but not KT framework) + Evidence to Policymaking + Agenda setting/ Policy change + LMIC + MH** | | | **(No framework, but tool/strategy) + KT + Evidence to Policymaking + LMIC + MH** | | |
| **n=0 identified** | **n=4 identified** | | | **n=6 identified** | | | **n=2 identified** | | | **n=13 identified** | | | **n=3 identified** | | | **n=2 identified** | | |
| **Publication / Framework** | **Publication** | **Frame-work** | **Publication** | | **Frame-work** | **Publication** | | **Frame-work** | **Publication** | | **Frame-work** | **Publication** | | **Frame-work** | **Publication** | | **Frame-work** |  |
| n.a. | Court, J., & Young, J. (2006). Bridging research and policy in international development: An analytical and practical framework. Development in Practice, 16(1), 85–90. | Context, evidence, links framework (RAPID) | Ashford, L. S., Smith, R. R., De Souza, R. M., Fikree, F. F., & Yinger, N. V. (2006). Creating windows of opportunity for policy change: Incorporating evidence into decentralized planning in Kenya. *Bull World Health Organ*, *84*(8), 669–672. | | Theorical framework for the transformation of knowledge to policy actions | Mulvale, G., Roussakis, C., Canning, C., Papadodoulos, D., & Knoops, F. (2017). Knowledge Mobilization and Mental Health Policy: Lessons from the Canadian Consensus Conference on the Mental Health of Emerging Adults. *Canadian Journal of Community Mental Health*, *36*(2), 1–41. | | Boyco 2012 Deliberate dialogues adapted | Flitcroft, K., Gillespie, J., Salkeld, G., Carter, S., & Trevena, L. (2011). Getting evidence into policy: the need for deliberative strategies? *Soc Sci Med*, *72*. | | Deliberati-ve models | Jenkins-Smith, H.C., Nohrstedt, D., Weible, C.M., & Sabatier, P.A. 2014. “The Advocacy Coalition Framework: Foundations, Evolution, and Ongoing Research.” In Theories of the Policy Process, Third Edition edited by Paul A. Sabatier and Christopher M. Weible. Boulder, CO: Westview Press, 183-223.  Sabatier 1987, 1988, 1993, 2003, 2006 | | Advocacy Coalition Framework (ACF) | Baingana, F., al’Absi, M., Becker, A. E., & Pringle, B. (2015). Global research challenges and opportunities for mental health and substance-use disorders. *Nature*, *527*(7578), S172–S177. | | Evidence-based strategies that accelerate the uptake of mental health research findings by policymakers |  |
|  | Jones, H., Jones, N., Shaxson, L., Walker, D., & Jones Nicola; Shaxson, Louise; Walker, David, H. J. (2013). Knowledge, policy and power in international development: a practical framework for improving policy. | Kowledge, policy and power (KPP) | Daniels, K., & Lewin, S. (2008). Translating research into maternal health care policy: a qualitative case study of the use of evidence in policies for the treatment of eclampsia and pre-eclampsia in South Africa. *Health Research Policy and Systems / BioMed Central*, *6*, 12. https://doi.org/10.1186/1478-4505-6-12 | | Kingdon's policy streams; Lavis et al 2002 (ideas, interests, institutions) | Goering, P., Butterill, D., Jacobson, N., & Sturtevant, D. (2003). Linkage and exchange at the organizational level: a model of collaboration between research and policy. *Journal of Health Services Research & Policy*, *8 Suppl 2*(suppl 2), 14–9. https://doi.org/10.1258/135581903322405126 | | Linkage and exchange | Contandriopoulos, D., Benoît, F., Bryant-lukosius, D., Carrier, A., Carter, N., Deber, R., … Lardeux, A. (2017). Structural analysis of health-relevant policy- making information exchange networks in Canada, 1–11. https://doi.org/10.1186/s13012-017-0642-4 | | Absorptive capacity assessment Framework on health-relevant policy-making information exchange networks in Canada (Planned project) | Petek, A., Novak, M., & Barry, M. M. (2017). Interdisciplinary research framework for multisectoral mental health policy development. International Journal of Mental Health Promotion, 19(3), 119–133. https://doi.org/10.1080/14623730.2017.1326398 | | Research framework of multisectoral mental health policy agenda-setting | Mackenzie J, Cassidy C. Global Mental Health Policy Influence Toolkit. 2015. | | Global Mental Health Policy Influence Toolkit |  |
|  | Redman, S., Turner, T., Davies, H., Williamson, A., Haynes, A., Brennan, S., … Green, S. (2015). The SPIRIT Action Framework: A structured approach to selecting and testing strategies to increase the use of research in policy. Social Science and Medicine, 136–137, 147–155. | SPIRIT Framework | El-Jardali, F., & Fadlallah, R. (2015). A call for a backward design to knowledge translation. *International Journal of Health Policy and Management*, *4*(1), 1–5. | | A conceptual framework for a backward design in Knowledge Translation (KT) |  | |  | Dobrow, M. J., et al. (2006). "The impact of context on evidence utilization: A framework for expert groups developing health policy recommendations." Social Science and Medicine 63(7): 1811-1824. | | Conceptual framework for context-based evidence-based decision-making (adapted from Dobrow et al., 2004). | Stockwell, A., Whiteford, H., Townsend, C., & Stewart, D. (2005). Mental health policy development: Case study of Cambodia. *Australasian Psychiatry*, *13*(2), 190–194. | | Combination of Walt and Gilson and Reich’s models for conceptual framework for mental health policy analysis in Cambodia |  | |  |  |
|  | Lavis, J. N., Lomas, J., Hamid, M., & Sewankambo, N. K. (2006). Assessing country-level efforts to link research to action. Bulletin of the World Health Organization, 84(8), 620–628. | Framework for assessing country-level efforts to link research to action | Hyder, A. A., Bloom, G., Leach, M., Syed, S. B., & Peters, D. H. (2007). Exploring health systems research and its influence on policy processes in low income countries. *BMC Public Health*, *7 (no pagi*(309). | | The research-policy interface in low-income countries |  | |  | Gold, M. (2009). Pathways to the use of health services research in policy. *Health Services Research*, *44*(4), 1111–1136. | | Pathways to the use of health services research in policy |  | |  |  | |  |  |
|  |  |  | Koon, A. D., Rao, K. D., Tran, N. T., & Ghaffar, A. (2013). Embedding health policy and systems research into decision-making processes in low- and middle-income countries. *Health Research Policy and Systems / BioMed Central*, *11*(30), 30. | | Conceptual framework for embeddedness in health research |  | |  | Hanney, S. R., & González-Block, M. A. (2009). Evidence-informed health policy: are we beginning to get there at last? *Health Research Policy and Systems*, *7*(1), 30. | | SUPPORT Tools for evidence-informed health Policymaking (STP) |  | |  |  | |  |  |
|  |  |  | Uzochukwu, B., Onwujekwe, O., Mbachu, C., Okwuosa, C., Etiaba, E., Nystrom, M. E., & Gilson, L. (2016). The challenge of bridging the gap between researchers and policy makers: Experiences of a Health Policy Research Group in engaging policy makers to support evidence informed policy making in Nigeria. *Globalization and Health*, *12 (1) (no*(67). | | Model  Four Evidence Informed Policy making strategies & Getting Research into Policy and Practice (GRIPP) Framework |  | |  | Hanney, S. R., Gonzalez-Block, M. A., Buxton, M. J., & Kogan, M. (2003). The utilisation of health research in policy-making: concepts, examples and methods of assessment. *Health Research Policy and Systems*, *1*, 2. | | Interfaces and receptors model |  | |  |  | |  |  |
|  |  |  |  | |  |  | |  | Haynes, A. S., Gillespie, J. A., Derrick, G. E., Hall, W. D., Redman, S., & Chapman, S. (2011). Galvanizers, guides, champions, and shields: the many ways that policymakers use public health researchers. *Milbank Q*, *89*. | | Patterns of usage/ Model for research(er) utilization |  | |  |  | |  |  |
|  |  |  |  | |  |  | |  | Hegger, I., Marks, L. K., Janssen, S. W. J., Schuit, A. J., Keijsers, J. F. M., & Oers, H. A. M. van. (2016). Research for Policy (R4P) : development of a reflection tool for researchers to improve knowledge utilization. *Implementation Science*, *11*(133), 1–10. | | “Research for Policy” tool |  | |  |  | |  |  |
|  |  |  |  | |  |  | |  | Lavis, J. N., Ross, S. E., Hurley, J. E., Hohenadel, J. M., Stoddart, G. L., Woodward, C. A., & Abelson, J. (2002). Examining the Role of Health Services Research in Public Policymaking. The Milbank Quarterly, 80(1), 125–154. | | Issue-based framework |  | |  |  | |  |  |
|  |  |  |  | |  |  | |  | Lomas, J., & Brown, A. (2009). Research and advice giving: a functional view of evidence-informed policy advice in a canadian ministry of health. *Milbank Q*, *87*. | | Linkage and exchange/ Functional framwork of push, pull and exchange |  | |  |  | |  |  |
|  |  |  |  | |  |  | |  | Moat, K. A., Lavis, J. N., & Abelson, J. (2013). How Contexts and Issues Influence the Use of Policy-Relevant Research Syntheses: A Critical Interpretive Synthesis. The Milbank Quarterly, 91(3), 604–648. | | Model how Contexts and Issues Influence the Use of Policy-Relevant Research Syntheses |  | |  |  | |  |  |
|  |  |  |  | |  |  | |  | Sauerborn, R., Nitayarumphong, S., & Gerhardus, a. (1999). Strategies to enhance the use of health systems research for health sector reform. *Tropical Medicine & International Health : TM & IH*, *4*(12), 827–835. | | Stakeholder-oriented model of policy-making |  | |  |  | |  |  |
|  |  |  |  | |  |  | |  | Wehrens, R. (2014). Beyond two communities - from research utilization and knowledge translation to co-production? *Public Health*, *128*(6), 545–551. | | Sheila Jasanoff’s5 co-production framework |  | |  |  | |  |  |
|  |  |  |  | |  |  | |  | Williams, I., & Bryan, S. (2007). Understanding the limited impact of economic evaluation in health care resource allocation: A conceptual framework. *Health Policy*, *80*(1), 135–143. | | Accessibility and acceptability |  | |  |  | |  |  |

In this review we identified 20 additional papers that were matching some of the inclusion criteria but not all, so they were not included in the results. An overview of all frameworks is presented in this table.
